# Pollinator parasites and the evolution of floral traits

**DOI:** 10.1002/ece3.4989

**Published:** 2019-05-15

**Authors:** Bertrand Fouks, Kaira M. Wagoner

**Affiliations:** ^1^ Department of Biology University of North Carolina at Greensboro Greensboro North Carolina

**Keywords:** behavioral immunity, floral evolution, host‐parasite interactions, plant–pollinator interactions, pollen dispersal, trait‐mediated indirect interactions, tripartite interactions

## Abstract

The main selective force driving floral evolution and diversity is plant–pollinator interactions. Pollinators use floral signals and indirect cues to assess flower reward, and the ensuing flower choice has major implications for plant fitness. While many pollinator behaviors have been described, the impact of parasites on pollinator foraging decisions and plant–pollinator interactions have been largely overlooked. Growing evidence of the transmission of parasites through the shared‐use of flowers by pollinators demonstrate the importance of behavioral immunity (altered behaviors that enhance parasite resistance) to pollinator health. During foraging bouts, pollinators can protect themselves against parasites through self‐medication, disease avoidance, and grooming. Recent studies have documented immune behaviors in foraging pollinators, as well as the impacts of such behaviors on flower visitation. Because pollinator parasites can affect flower choice and pollen dispersal, they may ultimately impact flower fitness. Here, we discuss how pollinator immune behaviors and floral traits may affect the presence and transmission of pollinator parasites, as well as how pollinator parasites, through these immune behaviors, can impact plant–pollinator interactions. We further discuss how pollinator immune behaviors can impact plant fitness, and how floral traits may adapt to optimize plant fitness in response to pollinator parasites. We propose future research directions to assess the role of pollinator parasites in plant–pollinator interactions and evolution, and we propose better integration of the role of pollinator parasites into research related to pollinator optimal foraging theory, floral diversity and agricultural practices.

## 1 INTRODUCTION

In plant–pollinator mutualisms, parasites (see Glossary) are of significant ecological and evolutionary importance (Bailes et al., 2018; Fouks & Lattorff, 2011; Fürst, McMahon, Osborne, Paxton, & Brown, 2014; reviewed in McArt, Koch, Irwin, & Adler, 2014). Parasites can weaken plant–pollinator mutualistic interactions, as they reduce mutual benefits for one or both partners, and can lead to conditional interactions in which outcomes depend on ecological conditions (Bronstein, 1994; Stanton, 2003). The threat of parasitism depends on the likelihood of parasite encounter and transmission, and is increased when multiple hosts share the same resource. Recent evidence demonstrates the major role of flowers in parasite transmission within and among pollinator species, with flowers acting as dispersal hot‐spots (Graystock, Goulson, & Hughes, 2015). While some plant–pollinator systems are exclusive, the vast majority of flowering plant species are visited by multiple species in a complex web of interactions (Fontaine, Dajoz, Meriguet, & Loreau, 2006; Goulson & Darvill, 2004). As a result, non‐host species (plant and pollinator) may also be important in pollinator–parasite interactions (McArt et al., 2014; Rigaud, Perrot‐Minnot, & Brown, 2010; Ruiz‐González et al., 2012). Moreover, plants are known to have adapted traits to reduce parasite burden, and therefore limit the loss of mutualistic benefits that result from parasitism (reviewed in McArt et al., 2014). Though many knowledge gaps remain, there is increasing evidence of the incidence and impact of parasite transmission among pollinator taxa (Bailes et al., 2018; Fürst et al., 2014; Graystock et al., 2013; McMahon et al., 2015). As pollinators facilitate the dispersion and transmission of flowering plant parasites, (reviewed in McArt et al., 2014), flowering plants facilitate the dispersion and transmission of pollinator parasites (Graystock et al., 2015; reviewed in McArt et al., 2014).

While parasites may not directly affect plant–pollinator interactions, they can impact those interactions indirectly. Indirect interactions have been shown to have important ecological (Hatcher, Dick, & Dunn, 2012; Wood & Johnson, 2015) and evolutionary consequences (Biere & Tack, 2013). These interactions can be divided into trait‐mediated and density‐mediated indirect interactions (Abrams, 1995, see Glossary). Density‐mediated indirect interactions have been a recent focus in plant–pollinator interactions due to pollinator decline (Biesmeijer et al., 2006; Potts et al., 2010). The global decline of pollinators has raised important concerns for human well‐being (Potts et al., 2016), since pollinators are vitally important to terrestrial ecosystems (Ashman et al., 2004) and to crop production (Klein, Steffan‐Dewenter, & Tscharntke, 2003). Moreover, the consequences of global pollinator decline on flowering plants have led to investigation of its impact on plant reproductive strategies and evolution (Thomann, Imbert, Devaux, & Cheptou, 2013). Density‐mediated indirect interactions are important in shaping ecological relationships, nevertheless it seems that trait‐mediated indirect interactions may be even more so (Schmitz, Krivan, & Ovadia, 2004). Parasites that change the behavior of prey or predator are thought to have keystone effect on community composition (Hatcher, Dick, & Dunn, 2014). While the impact of trait‐mediated indirect interactions has been the subject of numerous studies in plant–pollinator interactions (reviewed in Irwin, 2012), effects of pollinator parasites have been largely neglected. Studies regarding pollinator parasites have primarily focused on the effects of infection on pollinator foraging behavior (see Glossary), and few studies depict conflicting results on the impacts infection can have on their pollination services (reviewed in Koch, Brown, & Stevenson, 2017). While infected pollinators exhibit modified foraging behavior, pollinators have adapted a wide range of behaviors to defend themselves against parasites, which may be highly significant in shaping plant–pollinator interactions.

Behavioral defense mechanisms against parasitism are referred to as behavioral immunity, or the behavioral immune system (de Roode & Lefèvre, 2012; Hart, 2011; Schaller, 2011, see Glossary). Since it prevents or reduces parasitization without the costs of the immune system activation, behavioral immunity is a cost‐effective form of defense, and is thus widespread across the animal kingdom including many pollinator species (reviewed in de Roode & Lefèvre, 2012; Hart, 2011; Schmid‐Hempel, 2011). Such behavioral adaptations of pollinators in response to parasites could be of great importance for plant–pollinator interactions, as pollinator parasites can modify the value of a floral reward for pollinators and therefore the relative attractiveness of a flowering plant. Indeed, self‐medication in pollinators increases the attractiveness of flowering plants secreting nectar with secondary metabolites (Richardson, Bowers, & Irwin, 2016) and disease avoidance decreases the attractiveness of flowers harboring parasites (Fouks & Lattorff, 2011, 2013). Pollinator‐mediated selection, in theory, favors floral traits that maximize reproductive fitness via pollen export and import (Morgan, 1992). Thus many floral traits have evolved to attract pollinators (Fenster, Armbruster, Wilson, Dudash, & Thomson, 2004; Schiestl & Johnson, 2013) and to increase pollen transport (Kudo, 2003). In order for pollinator parasites to influence flower evolution, parasites must impact plant fitness by affecting pollen import and/or export, and plants must be able to adapt to reduce parasite impacts.

**Figure 1:**
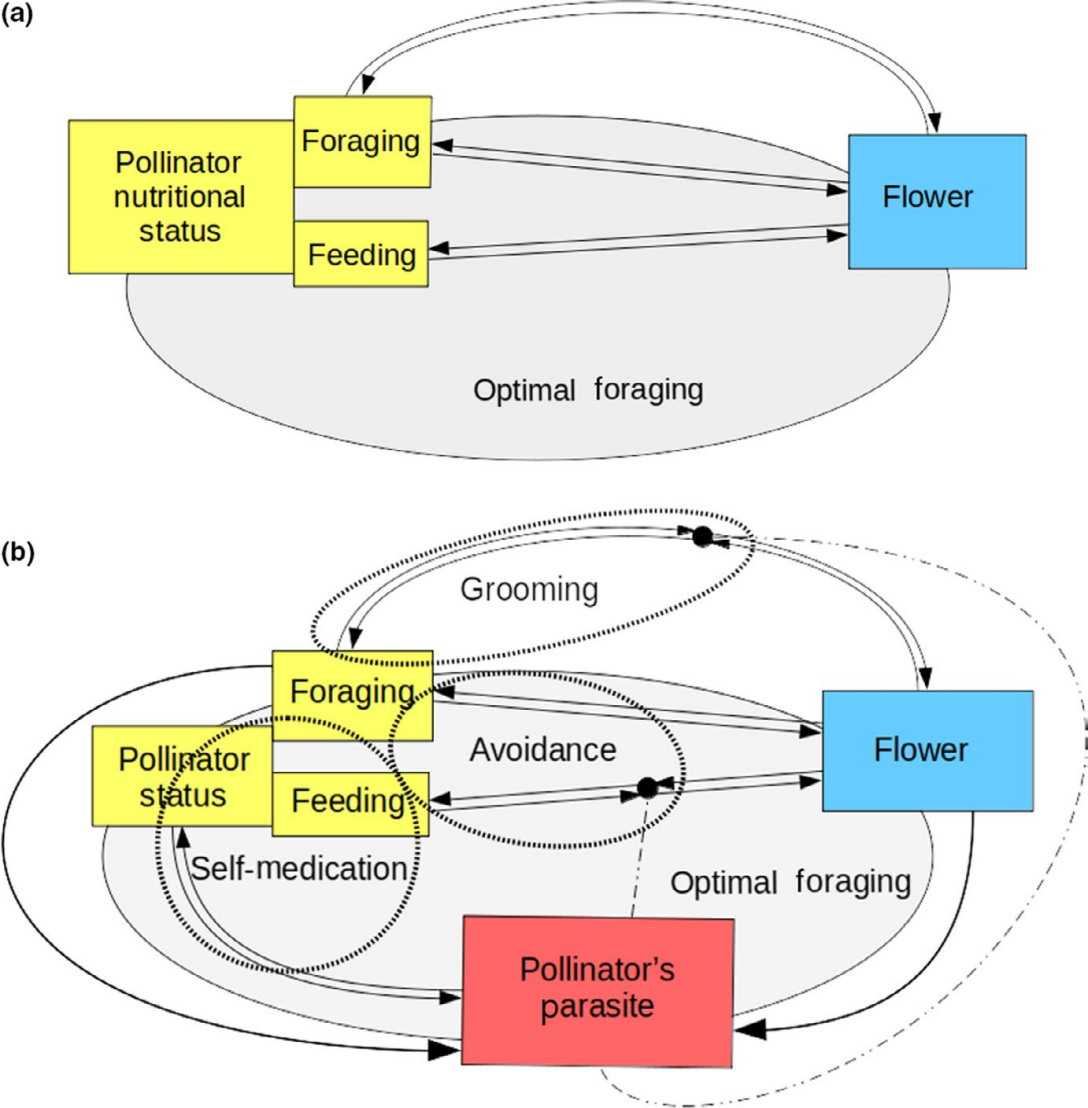
Conceptual diagram of plant–pollinator interactions without and with the impact of pollinator parasites (Box 1). (a) Plant‐pollinator interactions without pollinator parasites. Pollinators and plants interact through the foraging and feeding behaviors of pollinators. (b) Plant‐pollinator interactions with pollinator parasites (Box 1). The colors yellow, blue and red symbolize pollinators, flowers and pollinator parasites respectively. Two ways arrows represent species interactions, here either plant–pollinator or pollinator–parasite interactions. Impacts of pollinators and flowers on parasite transmission are represented as one‐way arrows. Dashed and dotted lines with a black dot symbolize disruption of plant–pollinator interactions by parasites. The black dots are surrounded by two arrows, symbolizing adaptation by both plants and pollinators to circumvent pollinator parasites disruption. The dashed ellipses delimit each pollinator immune behavior. The gray ellipse symbolizes the scope of pollinator optimal foraging

Despite recent evidence of the impact of parasites on plant–pollinator interactions (Richardson et al., 2016), the role of pollinator parasites on pollinator behaviors and floral evolution have not been thoroughly assessed. Here we discuss how self‐medication, disease avoidance, and immune grooming behaviors (see Glossary) have been adapted to defend pollinators against parasites, as well as how pollinator parasites, through these immune behaviors, can impact plant–pollinator interactions (Figure 1, Box 1). We further discuss how pollinator immune behaviors can impact plant fitness, and how floral traits may adapt to optimize plant fitness in response to pollinator parasites. Perspectives and future directions are proposed for further investigation of these complex interactions, and their potential role in the evolution of pollinators, their parasites, and plants.

Box 1 **Summary of the impact of pollinator parasites on plant–pollinator interactions**

1 Pollinator foraging and feeding behaviors have been optimized through plant–pollinator interactions, where floral traits respond to pollinators through adaptations that optimize plant fitness. Pollinator parasites induce modifications of plant–pollinator interactions and interact with pollinator status through infection. Infection modifies pollinator status, which can alter foraging behaviors (reviewed in Koch et al., 2017), driving foraging and feeding decisions (i.e., self‐medication). Pollinator parasite fitness is impacted by the foraging behavior of pollinators and by floral traits (Koch et al., 2017; McArt et al., 2014). Pollinators have adapted foraging and feeding immune behaviors to counter the impacts of pollinator parasites. Plants likely develop traits to optimize their fitness in response to pollinator immune behaviors. The behavioral adaptations of pollinators against parasites can be divided into 3 distinct behaviors: self‐medication, disease avoidance, and immune grooming. Self‐medication and disease avoidance result from both foraging and feeding adaptations of pollinators against the parasite threat, and are important factors for assessing pollinator optimal foraging strategies. Grooming is the result of pollinator foraging adaptations and may play an important role in pollen dispersal. However as it is a post‐feeding behavior, grooming may not significantly impact pollinator optimal foraging. Indeed, pre‐feeding foraging behaviors, such as patch searching and flower handling likely play a role in the optimization of pollinator foraging, while post‐feeding behaviors such as grooming and defecation have a lesser impact on pollinator foraging optimization, as they only modulate flower departure time.

## 2 THE UBIQUITY OF IMMUNE BEHAVIORS IN ANIMALS (AND POLLINATORS?)

Parasites are omnipresent in the environment (Thomas, Renaud, & Guégan, 2005) and represent a strong evolutionary force through reduction in life span and fitness of their host (Bonsall, 2004; Salathé, Kouyos, Regoes, & Bonhoeffer, 2008; Schmid‐Hempel, 2011). In response to this threat, animals have evolved a battery of defense mechanisms including many altered behaviors (Schmid‐Hempel, 2011). Almost all animals, from roundworms to humans (Curtis, 2014; Hart, 2011; Meisel & Kim, 2014; de Roode & Lefèvre, 2012; de Roode, Lefèvre, & Hunter, 2013), have evolved behavioral strategies to prevent and/or reduce parasite uptake, intake, establishment, spread and transmission. A wide variety of such behaviors have been adapted to reduce parasite threat (Hart, 2011; de Roode & Lefèvre, 2012). Here, we will focus on three common pollinator immune behaviors: self‐medication, disease avoidance, and immune grooming. Most existing evidence of pollinator behavioral immunity comes from bees. Nonetheless, it is highly likely that most pollinators, if not all, exhibit at least one of these immune behaviors while foraging. Self‐medication, long thought to be restricted to animals with high cognitive abilities, has been documented in invertebrates and can be the result of innate behaviors (de Roode et al., 2013). Disease avoidance is the first line of defense against parasitism and the most wide‐ranging immune behavior in animals (Hart, 2011; Schmid‐Hempel, 2011). Grooming is nearly universally represented in animal taxa and while it has diverse functions, its primary purpose is the removal of detritus and parasites (Sachs, 1988; Zhukovskaya, Yanagawa, & Forschler, 2013). Each of these pollinator immune behaviors has the potential to influence flowering plant fitness.

### 2.1 Self‐medication

Since nutrients, medicines, and toxins are often only differentiated by dose (Raubenheimer & Simpson, 2009), it is not surprising that chemicals utilized for self‐medication are typically already present in an individual's diet (reviewed in Erler & Moritz, 2015; Tritschler et al., 2017). The widespread availability of medicinal substances across plant species (reviewed Stevenson, Nicolson, & Wright, 2017) gives pollinators the opportunity to adapt their foraging decisions according to metabolite composition and concentration in response to parasite challenge. Multiple pollinators have been shown to self‐medicate to reduce the parasite burden in themselves, their offspring, and their nestmates (reviewed in de Roode & Lefèvre, 2012; Abbott, 2014). For example, honey bees incorporate antimicrobial resinous mixtures called propolis into their nests to reduce pathogen loads (reviewed in Simone‐Finstrom, 2017). Likewise, bumblebees have been shown to reduce parasitic infections using flower nectar containing alkaloids, terpenoid and iridoid glycoside (Manson, Otterstatter, & Thomson, 2010; Richardson et al., 2015).

### 2.2 Disease avoidance

Pollinators have been shown to reduce their feeding time on flowers harboring parasites, sometimes to the point of complete avoidance (Fouks & Lattorff, 2011, 2013). In addition to affecting flower visitation, parasite avoidance may have played a role in the evolution of alternative foraging strategies that facilitate consumption of flower nectar without flower visitation, as exemplified by nectar‐robbing (Box 2). Pollinator avoidance of flowers contaminated by parasites specific to the foraging pollinator species, and closely related pollinator species has been observed (Fouks & Lattorff, 2011, 2013; Yousefi & Fouks, 2019). Pollinator avoidance of parasites on flowers requires pollinator detection of cues indicating parasite presence. In the lab, bumble bees are known to negatively respond to floral bacteria odor cues depending on bacteria strains and density (Junker, Romeike, Keller, & Langen, 2014). Bumble bees have also been shown to rely on olfactory cues to recognize and avoid contaminated flowers when foraging (Fouks & Lattorff, 2011, 2013). Such olfactory cues could come from an interaction between parasites and the flower, or directly from the parasite. For example, microorganisms on flowers can modify nectar composition, including changes to sugar concentration and pH (Good, Gauthier, Vannette, & Fukami, 2014; Herrera, García, & Pérez, 2008; Vannette, Gauthier, & Fukami, 2013). However, bacteria are also known to synthesize a great diversity of volatiles on their own (Schulz & Dickschat, 2007), which may serve as olfactory cues of contamination to foraging pollinators. While olfactory cues may be the primary method by which pollinators detect parasites on flowers, parasite detection methods and abilities likely differ between pollinator species, and may be related to the floral cues species typically use to assess flower reward. For example, nectar and pollen‐foraging bees probe the flowers with their antenna (Evangelista, Kraft, Dacke, Reinhard, & Srinivasan, 2010; Lunau, Unseld, & Wolter, 2009) thereby gathering gustatory, olfactory and tactile information (Haupt, 2004; Kevan & Lane, 1985). In contrast, hummingbirds respond more strongly to compounds within nectar than those emitted as volatiles (Kessler, Gase, & Baldwin, 2008) and may therefore be unable to detect the presence of microbes in flowers without tasting nectar (Irwin, 2000). Visual cues likely play a primary role in detection of larger parasites, such as mites or fungal spores.

Box 2 **Nectar‐robbing by legitimate pollinators as a form of disease avoidance**

1 Nectar‐robbing has been observed in many species, including legitimate pollinators (reviewed in Irwin, Bronstein, Manson, & Richardson, 2010), and has been shown to impact plant fitness (Castro, Silveira, & Navarro, 2009; Irwin, 2006; Zhang, Yu, Zhao, & Guo, 2009; Zhang, Zhao, & Inouye, 2014). However, evidence indicates that the impact of nectar‐robbing on plant fitness varies widely, from no impact (Hazlehurst & Karubian, 2016), to significant negative (Burkle, Irwin, & Newman, 2007; Irwin & Brody, 1998) and positive (Singh, Barman, & Tandon, 2014) effects. While the benefits of nectar‐robbing represents for species that cannot access floral nectar are obvious, benefits for legitimate pollinators are less evident. The primary explanations for nectar‐robbing in legitimate pollinators are increased efficiency of nectar gathering compared to floral visits, and reduced competition between pollinators. In the context of pollinator parasites, high floral parasite presence could increase the frequency of nectar‐robbing as a method of reducing parasitization risk. Nectar‐robbing could allow pollinators to obtain floral resources without contacting contaminated flower regions, and thus has potential to be an immune behavior. In addition to a more efficient means of collecting nectar and avoiding competition, nectar‐robbing by legitimate pollinators could be favored by pollinator parasites. Either way, it would be interesting to investigate nectar‐robbing behavior in relation to the prevalence of pollinator parasites on flowers.

### 2.3 Immune grooming

All terrestrial animals display behaviors that are generally categorized as grooming (Sachs, 1988). Grooming is represented across a plethora of vertebrate and invertebrate taxa (Hlavac, 1975; Roy, Steinkraus, Eilenberg, Hajek, & Pell, 2006), and numerous instances of grooming as a defense against parasites have been documented (Zhukovskaya et al., 2013). While the impacts of floral parasite presence on pollinator grooming have never been studied, ants, bees, and termites are known to increase grooming behavior after exposure to parasites (Reber, Purcell, Buechel, Buri, & Chapuisat, 2011; Traniello, Rosengaus, & Savoie, 2002; Westhus et al., 2014; Wilson‐Rich, Spivak, Fefferman, & Starks, 2009). Given the prevalence of the behavior, it is likely that bees groom intensively after encountering parasites on flowers, just as they do after infection with parasites acquired from other sources (Peng, Fang, Xu, & Ge, 1987; Sammataro, Gerson, & Needham, 2000).

## 3 DO POLLINATOR IMMUNE BEHAVIORS IMPACT PLANT FITNESS?

### 3.1 Self‐medication

Despite numerous examples of self‐medication in pollinators (Abbott, 2014; de Roode & Lefèvre, 2012), evidence of self‐medication during pollination is currently limited to bumblebees. Infected bumble bees feed longer and are more likely to forage a second time on flowers with high concentrations of iridoid glycosides (Richardson et al., 2016). Moreover, plants with high concentrations of iridoid glycosides display higher pollen transfer to conspecifics than plants with low concentrations of iridoid glycosides (Richardson et al., 2016). While it is unclear whether this was a result of increased visitation due to self‐medication (Richardson et al., 2016), changes in pollinator visit number and duration are known to have significant effects on plant fitness (Ivey, Martinez, & Wyatt, 2003; Mitchell & Waser, 1992; Sahli & Conner, 2007).

### 3.2 Disease avoidance

While there is no direct evidence of the impact of floral parasite presence on plant fitness, parasite presence on flowers seems to reduce the relative attractiveness of their reward (Fouks & Lattorff, 2011, 2013). Moreover, pollinator disease avoidance can lead to a reduction in the overall pollinator visitation rates (Yousefi and Fouks, 2019). Since reduced pollinator visitation leads to pollen limitation (Knight et al., 2005) and decreased plant reproductive success (Irwin & Brody, 1998), the presence of pollinator parasites on flowers is likely to negatively impact plant fitness. In addition, the positive correlation between pollinator visit duration and plant fitness (Ivey et al., 2003) suggests that reduced pollinator visit duration on parasite‐contaminated flowers may lead to reduced plant fitness. In *Mimulus aurantiacus*, presence of bacteria but not yeast in the nectar resulted in decreased pollination success and a reduction in seed set (Vannette et al., 2013).

One might object that for pollinator parasites to be present on flowers, they need to be deposited by pollinators, which may result in flower pollination. Nevertheless, one pollinator visit may not be sufficient for a successful pollination. Furthermore after the deposition of pollinator parasites, the subsequent avoidance of the flower by pollinators may lead to a reduced pollen dispersal, as many flowers are hermaphrodites. In addition, most flowering plant species are visited by multiple pollinator species (Fontaine et al., 2006) and not all pollinator species provide efficient pollination service (Koski, Ison, Padilla, Pham, & Galloway, 2018). Therefore, it is possible that pollinator parasites can be deposited by inefficient pollinators and consequently lead both inefficient and efficient pollinators to avoid contaminated flowers. In such scenario, plant fitness may drastically be reduced, as both female and male fitness will be impacted.

### 3.3 Immune grooming

**Figure 2:**
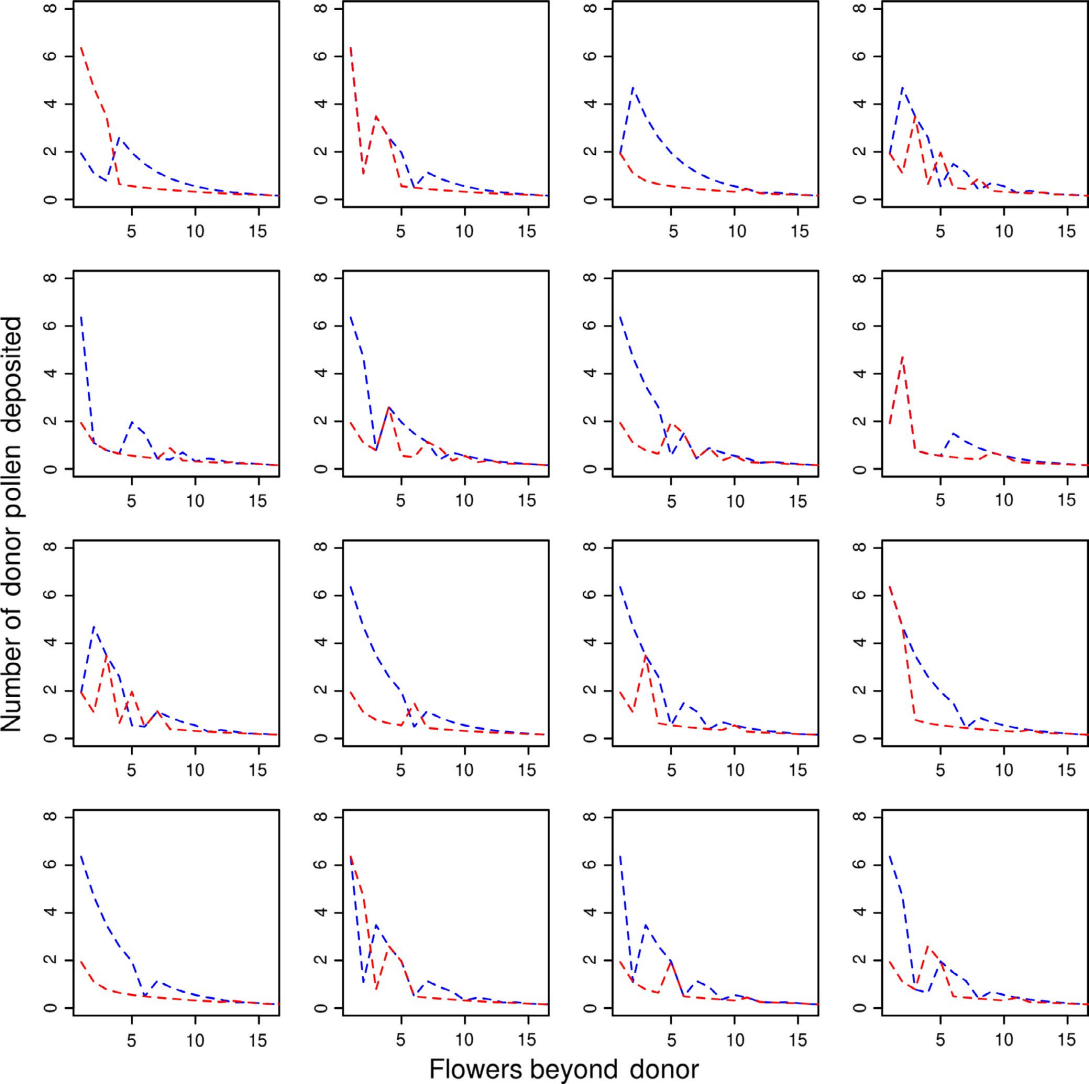
Pollen dispersal of 16 simulations in relation to different parasite prevalence rates and order of foraging on contaminated flowers. These simulations reveal the importance of the contamination status of the first flowers (rather than overall parasite prevalence) in pollen dispersal. The red dashed line represents a floral parasite prevalence of 80%, while the blue line represents 20% parasite prevalence. *R* = 100, *π*_s_ = 0.05, *π*_e_ = 0.45, *π* = 0.5, *y*_s_ = 0.1, *ρ*_e_ = 0.2, *ρ*_s_ = 0.1, noncontaminated flowers: Г = 0.1, contaminated flowers: Г = 0.6

**Figure 3:**
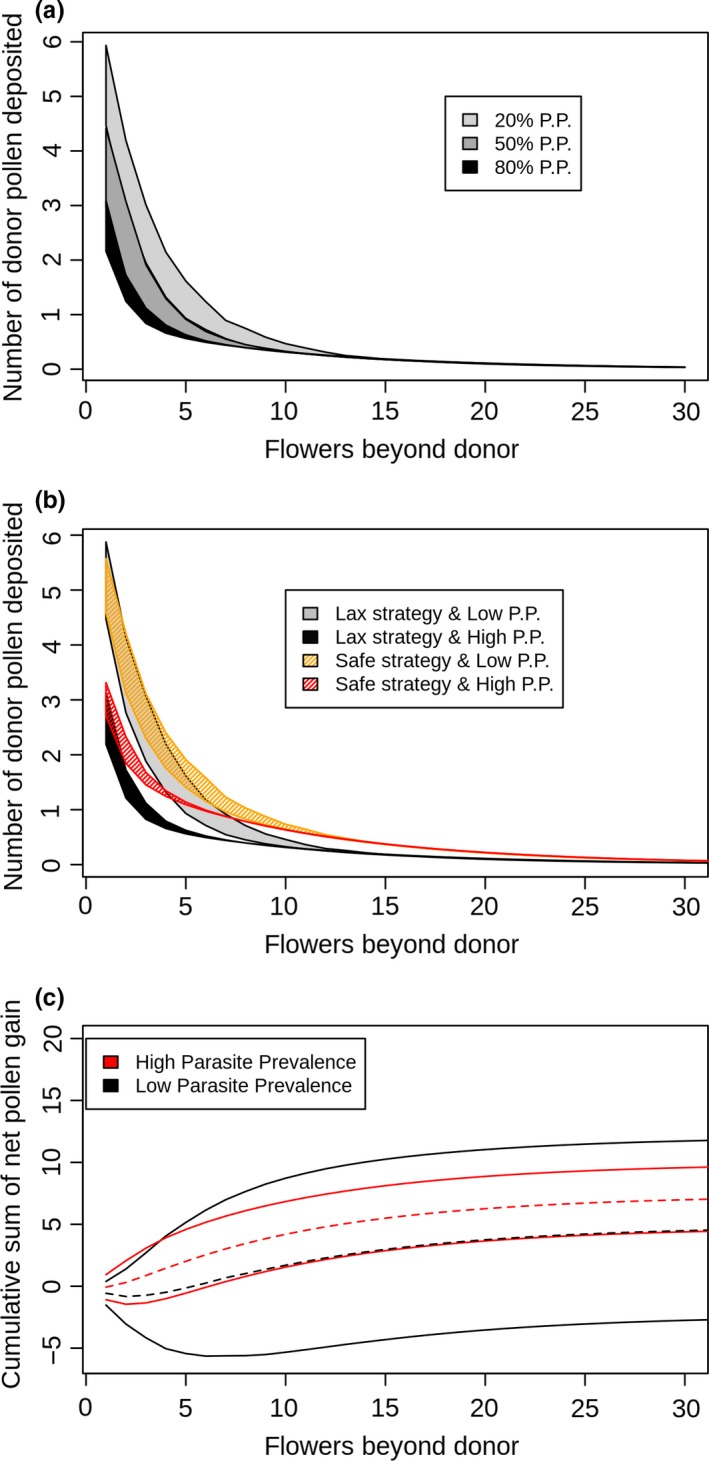
Pollen dispersal models. (a) Model of pollen dispersal in relation to parasite prevalence on flowers. (b) Model of pollen dispersal in relation to parasite prevalence on flowers and flower pollen dispersal strategies. (c) Model of differential cumulative net pollen gain (difference in total amount of pollen donated to flowers beyond donor) between flower strategies with high (red lines) and low parasite prevalence (black lines). The solid lines represent the minimum and maximum of cumulative pollen gain, and the dashed lines represent the mean pollen gain. Values below 0 indicate that the *lax strategy* leads to more pollen deposited to subsequent flowers, while values above 0 demonstrate higher pollen deposition for the *safe strategy*. Our model demonstrates the impact of floral parasite prevalence on pollen dispersal, and suggests that the benefits of the adaptation of floral traits optimizing pollen dispersal are highly dependent on overall floral parasite prevalence on flowers. P.P. = Parasite Prevalence, high parasite prevalence: 80%, low parasite prevalence: 20%. *R* = 100, *π*_s_ = 0.05, *π*_e_ = 0.45, *π* = 0.5, *y*_s_ = 0.1, *ρ*_e_ = 0.2, *ρ*_s_ = 0.1, noncontaminated flowers: Г = 0.1, contaminated flowers: Г = 0.6, safe strategy: *π*_e_ = 0.35, *π*_s_ = 0.15; 70/30, and lax strategy: *π*_e_ = 0.45 and *π*_s_ = 0.05; 90/10

Pollinators primarily groom in order to gather pollen as a food source (Harder, 1990). It is generally understood that grooming reduces pollen dispersal and that the reduction in pollen carryover depends on the timing and intensity of grooming (Castellanos, Wilson, & Thomson, 2003; Harder & Wilson, 1998; Rademaker, de Jong, & Klinkhamer, 1997; Thomson, 1986). Grooming immediately following removal of pollen from a donor flower should reduce pollen carryover considerably because the largest loads of pollen from a particular donor are usually deposited on the first few recipient flowers (Castellanos et al., 2003; Rademaker et al., 1997; Thomson, 1986). Most pollen‐foraging bees groom to some extent after every flower visited, packing most of the removed pollen into their corbiculae, which reduces the amount of pollen available for transfer to stigmas. These grooming events vary considerably in relative intensity, with the intensity and frequency of grooming increasing as bees accumulate pollen on their bodies during foraging (Harder, 1990). Grooming behavior and its influence on pollinator‐mediated gene dispersal have primarily been studied in bees (Holmquist, Mitchell, & Karron, 2012; Thomson, 1986). Bees are known to groom when infested with mites (Peng et al., 1987; Sammataro et al., 2000), and may therefore increase their grooming intensity when encountering parasites on flowers. Theoretically, the presence of pollinator parasites on flowers could intensify pollinator grooming and thus drastically influence pollen dispersal (Box 3, Figures 2 and 3a).

Box 3 **Pollen dispersal model in relation to floral pollinator parasites presence.**

1 We modeled pollen dispersal by a pollinator from one donor flower to consecutively visited flowers using the two‐compartment mathematical model developed by Harder and Wilson (1998). In this model, the flower contains R pollen grains which are available for pollinators to pick up. Pollinators pick up pollen either on safe sites of their body at the rate of *π*_s_ or on exposed sites of their body at the rate of *π*_e_. Pollen in exposed sites of a pollinator's body has two non‐mutually exclusive fates: a fraction of pollen can be deposited on recipient stigmas (*ρ*_e_) or pollen can be displaced. Displaced pollen is either moved to safe sites of a pollinator's body at a rate of Г*y*_s_ or lost at a rate of Г*L*, where Г = grooming intensity. Pollen in safe areas of a pollinator's body is deposited on recipient stigmas at a rate of *ρ*_s_. Using this model, pollen dispersal across different grooming intensities was plotted (Supporting Information Figure S1a). As illustrated above, high grooming intensity leads to a rapid depletion of pollen, resulting in a small percentage of donor pollen being dispersed to the next five flowers visited by the pollinator. After the 10th consecutive flower visited by the pollinator, pollen deposition does not differ significantly between pollinators exhibiting high and low grooming intensities. This model incorporates various grooming intensities, however in our case we expect to have only high intensity of grooming when flowers are contaminated by a parasite (intensive grooming as immune behavior). The pollen dispersal of a donor flower by a pollinator was then calculated as a function of flower contamination (Figure 2). Most of the pollen is deposited or lost on the first five flowers visited by the pollinator, regardless of overall parasite prevalence. Thus, the contamination status of the first five flowers is decisive for the fate of most pollen. Due to this variation of pollen dispersal depending on the contamination status of the first flowers, the pollen dispersal was averaged (on 100 random runs) between different flower orders bearing pollinator parasites of various quantities. The highest and lowest average of pollen deposited on each flowers were drawn (from 1,000 averages) in relation to parasite prevalence (Fig. 3a). Parasite prevalence on consecutive flowers affects the magnitude of pollen dispersal (as illustrated in Supporting Information Figure S1a). The presence of pollinator parasites on flowers could hinder pollen dispersal if pollinators exhibit increased grooming as a response to parasites. It is possible that flowers have adapted different strategies for pollen dispersal in the presence of pollinator parasites. For example, some morphological floral traits could be selected to increase the exposure of pollen to safe sites on the pollinator's body. Here, we assumed that *π*_e_ and *π*_s_ are the proportion of the overall *π* pollen picked up by pollinators, where *π* is a constant. Flowers could adapt a longer anther (*safe strategy*:* π*_e_ = 0.35, *π*_s_ = 0.15; 70/30) to increase the number of pollen grains deposited on safe sites of pollinators. For flowers without a long anther (*lax strategy*:* π*_e_ = 0.45 and *π*_s_ = 0.05; 90/10) we modeled a higher rate of *π*_e_ with the same overall *π* (Supporting Information Figure S1b). While the safe strategy for a flower increases its pollen dispersal when there is high grooming intensity, this benefit is less obvious when grooming intensity is low (Supporting Information Figure S1b). In the same manner, high parasite prevalence makes the safe strategy of a flower more beneficial for pollen dispersal than lax strategy flowers (Figure 3b). However, when parasite prevalence is low, this strategy does not significantly increase the number of pollen grains dispersed. The net cumulative gain of pollen dispersed (total number of pollen donated to flowers beyond the donor) was accounted for using the minimum and maximum difference in pollen dispersed between the two strategies. According to the model, the safe strategy is only beneficial in environments with high parasite prevalence (Figure 3c). Variation of pollen dispersal (due to the order of contamination status on following flowers) demonstrates that the safe strategy of flowers in environments of low parasite prevalence is highly variable, and can be negative for pollen dispersal compared to a flower with lax strategy (Figure 3c). This model demonstrates that the presence and high prevalence of pollinator parasites on flowers favor the adaptation of long anthers or other traits similarly capable of increasing the proportion of pollen deposited on safe sites of pollinator bodies.

## 4 CAN FLORAL TRAITS MODULATE POLLINATOR IMMUNE BEHAVIORS TO OPTIMIZE PLANT FITNESS?

### 4.1 Self‐medication

While secondary plant metabolites likely evolved as a form of herbivore resistance (Strauss & Whittall, 2006), their presence in floral nectar is puzzling as nectar is primarily used by pollinators. Indeed, secondary plant metabolites in nectar can be repellent for pollinators (Adler, 2000; Adler & Irwin, 2005). However, their presence can be beneficial for plants by facilitating specialization in plant–pollinator interactions through the protection of nectar from nectar‐robbing, inhibition of microbial growth, preservation of beneficial nectar microbiomes, and attraction of self‐medicating pollinators (reviewed in Stevenson et al., 2017). In synergy with other selective forces, pollinator self‐medication may drive the evolution of flowering plants containing nectar with non‐negligible concentrations of diverse secondary metabolites.

The decline of many pollinator species has been reported (Lever, van Nes, Scheffer, & Bascompte, 2014; Potts et al., 2010; Tepedino, Durham, Cameron, & Goodell, 2015), and parasitism seems to be an important factor contributing to pollinator loss (Goulson, Nicholls, Botías, & Rotheray, 2015; Potts et al., 2010). Recent evidence suggests that the dependence of honey bees on disappearing medicinal plants may partly explain honey bee colony losses (Tihelka, 2017). In addition, the wide use of domesticated pollinators and commercialized insect pollinators (i.e., honey bees and bumble bees) leads to the transmission and spread of parasites to wild pollinator populations (Gisder & Genersch, 2017; Goulson & Hughes, 2015; Murray, Coffey, Kehoe, & Horgan, 2013; Otterstatter & Thomson, 2008; Whitehorn, Tinsley, Brown, & Goulson, 2013). In this context, one might predict the attractiveness of plants that produce nectar with relatively high concentrations of secondary metabolites to increase in global pollinator populations.

### 4.2 Disease avoidance

Plants may adapt floral traits to decrease the negative impacts of pollinator parasites and related avoidance. Such traits can be divided into three main categories: (a) floral traits that diminish parasite deposition on flowers and transmission to visiting pollinators (reviewed in McArt et al., 2014), (b) floral traits that affect behavioral avoidance of pollinators, and (c) floral traits that reduce pollinator parasite loads and viability. Parasites can be deposited and picked up at different rates depending in part on flower shape (Durrer & Schmid‐Hempel, 1994; Graystock et al., 2015). Parasite transmission can be affected by floral traits that affect physical contact and/or flower handling time during foraging (reviewed in McArt et al., 2014). Some parasite dispersal may be a result of spore adhesion to the bee cuticle, and subsequent delivery of spores to other surfaces (Graystock et al., 2015). Recent evidence suggests that flower architecture may play a role in pollinator disease avoidance (Yousefi and Fouks, 2019). Indeed, flower species with architectures limiting the floral surface in contact with visiting pollinators were not affected by the deposition of larval honey bee parasites (Yousefi and Fouks, 2019). In addition, avoidance rate has been shown to be dependent on both plant and pollinator species, suggesting the role of flower handling in pollinator avoidance and parasite transmission (Durrer & Schmid‐Hempel, 1994; Yousefi and Fouks, 2019).

Pollinator parasites may favor the adaptation of floral traits such as scent, nectar volume and nectar composition in order to increase flower attractiveness, and reduce the detection of parasites by pollinators. In bumble bees, the presence of floral scent on artificial flowers impairs the pollinators ability to avoid contaminated flowers (Fouks, Robb, & Lattorff, 2019), likely due to reduced olfactory detection. Some plant species possess scented nectar which may favor plant fitness through pollinator attraction, deterrence of nectar robbers and florivores, antimicrobial activity, and communication with predators and parasitoids (Raguso, 2004). In addition, scents in nectar may favor plant fitness by diminishing the negative effect of pollinator parasites on pollinator visit frequency and duration.

Finally, flowers may adapt to reduce parasite load through the production of antimicrobial compounds (Nicolson & Thornburg, 2007), such as those often associated with floral scents (Raguso, 2004). While plants can reduce the presence and limit growth of microbes in their nectar through the production of antimicrobial compounds, there is no evidence that they are able to limit growth of pollinator parasites, which are usually deposited on flowers as spores, and need a host to complete their reproductive cycle. However, the presence of secondary metabolites in nectar may reduce the viability of pollinator parasites and the likelihood of pollinator infection, as secondary metabolites can reduce pathogen growth within the host (Manson et al., 2010).

### 4.3 Immune grooming

The “two‐compartment” pollen dispersal model developed by Harder and Wilson (1998) stipulates that donor pollen can either be deposited on “safe sites” or “exposed sites” of the pollinator's body. While all pollen deposited on the “safe sites” of the pollinator have the potential to reach a stigma, the pollen on “exposed sites” can be deposited on stigmas, lost, or moved to “safe sites”. Here we offer a modified version of the “two‐compartments” model, which includes conditional increases in grooming intensity depending on floral parasite presence, mimicking immune grooming (Box 3). This modified model allows an improved understanding of the potential effects of floral parasite presence on pollen dispersal. In the model, the likelihood of pollen dispersal is directly affected by the contamination status of the first few flowers visited by the pollinator (including the contamination status of the donor flower), regardless of parasite prevalence (Figure 2). Variation in pollen dispersal is therefore accounted for in the model with respect to varying degrees of parasite prevalence (Figure 3a). Here, the two‐compartments model was also modified to account for the benefits of different flower strategies of pollen dispersal for varying degrees of parasite prevalence. Two alternatives have been modeled: the *lax strategy,* where most of the pollen reaches “exposed sites”, and the *safe strategy,* where a higher proportion of pollen reaches “safe sites”. The different pollen dispersal strategies are more or less efficient, depending on parasite prevalence (Figure 3b). The *safe strategy* lead to a constant higher pollen dispersal compared to the *lax strategy* only when there is high parasite prevalence (Figure 3c). This result illustrates that, in the case of immune grooming, floral traits adaptations that increase the amount of pollen deposited on “safe” parts of pollinators (e.g., long anther) will increase plant fitness only when parasites are highly prevalent on flowers.

## 5 FUTURE DIRECTIONS

Most of our understanding of pollinator behavioral immunity and its impact on plant–pollinator interactions comes from indirect evidence in bees (Fouks & Lattorff, 2011; Richardson et al., 2016). Direct evidence of the impact of pollinator parasites on plant fitness needs to be gathered and should be extended to other pollinator species. In addition, examples of the selection of floral traits against pollinator parasites need to be sought after and identified. The experiment from Gervasi and Schiestl (2017) represents a powerful tool for gathering direct evidence of the adaptation of floral traits in response to different selective forces. In this experiment, fast cycle *Brassica rapa* plants were pollinated over 11 generations by either bumblebees, hover‐flies or hand‐pollination and several plant traits were measured (e.g., plant height, petal sizes, floral scent, and nectar volume). This methodology facilitated demonstration of the effects of differential selection imposed on plants, including the plants ability to rapidly diverge in response to different pollination vectors. This experiment could easily be adapted to evaluate the selective forces related to each pollinator immune behavior on floral traits. Such experiments would provide insight regarding the strength of the role pollinator parasites play in floral evolution. Follow‐up experiments could incorporate additional factors (e.g., herbivory), facilitating an evaluation of how complex interactions among multiple different factors drive floral evolution.

### 5.1 Self‐medication

While the attention paid to self‐medication by pollinators has been growing in recent years, (reviewed in Koch et al., 2017; Stevenson et al., 2017), the study of plant adaptation in the context of self‐medicating pollinators is still lacking. Flowers can differ in their nectar composition within a single plant, within a population, and among populations, as nectar amino‐acids are largely affected by environmental factors (reviewed in Nicolson & Thornburg, 2007). Parasitized pollinators are known to increase over the pollinating season, with the highest proportion of infected pollinators occurring in the middle of the summer (Imhoof & Schmid‐Hempel, 1999; Popp, Erler, & Lattorff, 2012; Strauss et al., 2013). Though it is yet to be demonstrated, seasonal increases in parasite prevalence would likely lead to corresponding increases in the proportion of self‐medicating pollinators. As such, plants with nectar containing high concentrations of relevant secondary metabolites can be expected to benefit from higher visitation rates during this period. Considering that such seasonal variation in pollinator parasite prevalence is constant over years, plants used medicinally by pollinators may be able to increase their fitness by adapting their flowering time (phenology) to match high parasite prevalence in relevant pollinators. Therefore, seasonal screening for pollinator parasites in a natural environment could be used to determine whether a relationship between flowering time of medicinal plants and parasite prevalence exists. Such a relationship would indicate that floral phenological adaptation can optimize pollinators’ attraction.

In a broader context, pollinator self‐medication could impact entire plant communities. Co‐flowering plant species can influence each other through indirect effects, such as shared pollinators (Carvalheiro et al., 2014). Depending on floral traits similarity, flower abundance and accessibility, shared pollinators may lead co‐flowering plant species to either facilitation or competition (Carvalheiro et al., 2014). Pollinator self‐medication could facilitate co‐flowering between plant species with nectar possessing different secondary plant metabolites concentrations. High concentration of secondary plant metabolites can be repellent for healthy pollinators, which may limit competition between plant species as healthy pollinators are likely to preferentially forage on plant with low secondary plant metabolite concentrations. In addition, plant species producing high concentrations of secondary plant metabolites may reduce the prevalence of pollinator infection such that pollinator diets shift back to nectar with lower concentrations. To understand the impact of pollinator self‐medication in co‐flowering between plant species, fitness of and competition between plants in response to pollinator self‐medication could be assessed through the controlled pollination of plants with varying degrees of medicinal properties by pollinator populations with varying parasite loads.

### 5.2 Disease avoidance

A thorough investigation of the abilities of pollinators to detect parasites is crucial to improving our understanding of pollinator avoidance behavior. In *Drosophila melanogaster*, a conserved olfactory circuit dedicated to avoidance of pathogenic *Penicillium* mold and *Streptomyces* soil bacteria has been identified (Stensmyr et al., 2012). Though such observations are still lacking in pollinators, the advances of genomics and sequencing of several pollinator genomes (Clare, Schiestl, Leitch, & Chittka, 2013; Haddad et al., 2018; Sadd et al., 2015) may allow the identification of similar pathways that are conserved among pollinators. The use of Proboscis extension response experiments could help identify those pathways, as has been done with toxic nectar compounds (reviewed in Stevenson et al., 2017). Experiments described in Riffell, Lei, Abrell, and Hildebrand (2013); Riffell et al. (2014) combine gas‐chromatography mass‐spectrometry, electro‐antennography and pollinator conditioning to detect volatile organic compounds of flowers and their effect on moth neural activation and learning. Modifications of these experiments may allow identification of volatiles involved in the detection of parasites by pollinators, as well as identification of the neurobiological mechanisms involved in such detection.

Future studies related to pollinator parasite avoidance should also assess the effects of floral architecture on disease avoidance as a function of pollinator and parasite species. For example, an extension of the experiment from Graystock et al. (2015) could help determine which floral traits impact pollinator parasite transmission and pollinator disease avoidance. Such information could be sought by testing the efficiency with which one pollinator species disperses parasites on flowers possessing various architectural characteristics, and recording the subsequent transmission of parasites through pollinator populations, as well as the degree to which contaminated flowers are avoided.

Pollinators are typically flexible in resource use (Brosi & Briggs, 2013; Fründ, Dormann, Holzschuh, & Tscharntke, 2013; Inouye, 1978), allowing for plasticity in ecological network topology (Spiesman & Gratton, 2016). At the community scale, this flexibility affects interactions, and may lead to species turnover (Simanonok & Burkle, 2014; Trøjelsgaard, Jordano, Carstensen, & Olesen, 2015). The presence of pollinator parasites on flowers could enhance the flexible foraging of pollinators, and influence pollinator species shifts. Despite flexible foraging, pollinators do not forage on all available flowers. Instead, pollinators demonstrate general preferences for some flower species, and typically limit pollen collection to a single species during each foraging bout (Waser, 1986). This specialization is beneficial for plants, as it reduces hetero‐specific pollen transfer, and may have been adapted by pollinators in order to limit the spread of parasites (Spiesman & Gratton, 2016). It would therefore be interesting to combine plant–pollinator and pollinator–parasite networks to assess the importance of pollinator parasites on plant–pollinator network topology. Taking parasite prevalence into account when evaluating plant–pollinator ecological networks could improve the predictability of plant–pollinator network topology and resilience.

### 5.3 Immune grooming

The effects of the presence of floral pollinator parasites on pollen dispersal are largely unknown, and thus represent a significant gap in our current understanding of pollinator–plant interactions. Future studies should directly assess grooming intensity of pollinators after exposure to contaminated and uncontaminated flowers. If pollinator parasites acquired during foraging trigger grooming, an assessment of grooming intensity and related effects on plant fitness should be conducted for plants with varying mating strategies. Such studies could significantly improve our understanding of the effects of pollinator parasites on plant mating strategies. The mathematical models presented here should be evaluated and/or improved through comparisons with empirical data regarding pollen dispersal in flowers with different out‐crossing strategies. For example, in heterostylous species (those with a simply inherited sexual polymorphism (reviewed in Barrett, 2002)), populations are composed of two (distyly) or three (tristyly) floral morphs that differ reciprocally from one another in the positions in which anthers and stigmas (where pollen is deposited by pollinators) are located in flowers. As shown in our pollen dispersal model, one or another morph could be favored depending on parasite prevalence on flowers. As parasite prevalence is not likely to be constant over space and time, immune grooming may contribute to the maintenance of such sexual polymorphism in plants.

## 6 PERSPECTIVES

### 6.1 Integrating pollinator behavioral immunity into optimal foraging theory

Lozano (1991) first recommended incorporation of the effects of parasites into optimal foraging theory, a behavioral ecology model that predicts animal foraging patterns to be selected to maximize fitness. Since then, numerous studies have demonstrated theoretical (Poissonnier, Lihoreau, Gomez‐Morachob, Dussutour, & Buhl, 2017; Ponton, Wilson, Cotter, Raubenheimer, & Simpson, 2011) and observational (Fouks & Lattorff, 2011; Tritschler et al., 2017) evidence of the significant role parasites play in trophic interactions. However, these studies have primarily investigated behavioral immunity through self‐medication, largely neglecting the effects of the avoidance of contaminated food sources. Further integration of self‐medication and avoidance behaviors into optimal foraging theory is critical for the much‐needed evaluation of the impacts of pollinator parasites on plant–pollinator interactions (Figure 1, Box 1). Immune grooming could be also added to the optimal foraging model, however its impact on foraging will likely be limited, since it only affects time until departure after feeding. In contrast, avoidance directly impacts foraging decisions. Fouks and Lattorff (2013) demonstrate that the presence of parasites in nectar leads pollinators to forage on those flowers as they would on flowers with low food rewards. Parasite presence in nectar could therefore be modeled similarly to a decreased nectar reward. When parasites are present, pollinators likely balance the risk of infection with the potential benefits associated with nectar quantity and quality. Therefore, under optimal foraging theory, pollinators should adapt their foraging behavior to maximize resource intake while minimizing parasite infection. Such optimization could lead not only to a binary choice between feeding and avoiding, but also to a modulation of feeding time, and the development of alternative feeding strategies (Box 2). There are numerous parameters a pollinator needs to assess to optimize its foraging strategy in response to floral parasite presence, which should lead to complex pollinator decision‐making. The cost of foraging on contaminated flowers likely varies according to the pollinator's existing infection status, the likelihood of parasite transmission, and the parasite virulence. In order to predict the extent of the impact of pollinator parasites on plant–pollinator interactions, a better understanding of the complex modulation of pollinator foraging strategies in response to parasites is needed.

### 6.2 Can pollinator behavioral immunity favor floral diversity?

The ecological impacts of parasites are complicated and are known to both positively and negatively affect biodiversity, depending on parasite specificity and environmental variables. Parasites, however, are rarely a major driver toward species extinction (reviewed in Hatcher et al., 2012). The activation of immune behaviors in pollinators depends on the pollinator, parasite, and plant species, as well as the parasite prevalence and multiple related interactions. The flexibility of plant–pollinator interactions favors floral diversity through balancing selection. Moreover, pollinator parasites (in addition to other factors such as competition) may drive the adaptation of complex pollinator foraging behaviors, and pollinators likely rely on several signals and cues to precisely assess floral contamination status. Complex foraging behaviors likely drive floral trait adaptation in order to maintain pollinator mutualisms (Bronstein, 1994; Stanton, 2003). In plants, several floral traits are known to influence the transmission of both plant and pollinator parasites (reviewed in McArt et al., 2014). The adaptation of multiple traits in response to one selective force may enhance the release of other traits from antagonistic selection pressures (Ehrlich & Raven, 1964), and may therefore simultaneously optimize plant fitness against several selection forces. To optimize foraging behavior, pollinators need to take into account the risk of parasite exposure during flower visitation, as well as several other factors such as quantitative and qualitative resource intake, predation risk, and competition (Biernaskie, Walker, & Gegear, 2009; Dawson & Chittka, 2014; Fouks & Lattorff, 2011; Leadbeater & Chittka, 2009). Therefore multi‐modal communication is likely necessary for pollinators to optimally exploit their complex environments (Leonard, Dornhaus, & Papaj, 2011). Further understanding of the role of pollinator immune behaviors on plant–pollinator interactions is needed to assess the impact of pollinator parasites on the diversity of plant–pollinator communities.

### 6.3 Behavioral immunity and land‐use change

Detailed investigation of the effects of pollinator parasites on plant–pollinator interactions could help improve our understanding of the drivers of global pollinator decline (Potts et al., 2010). Such decline could be influenced by the disappearance of the medicinal plants on which pollinators depend (Tihelka, 2017). In addition, mass‐flowering crops lead to reduction in pollinator abundance (Holzschuh et al., 2016), perhaps in part by facilitating the transmission of parasites between pollinators. Monocultural practices dominate much of modern agriculture. As floral architecture is important for pollinator parasite dispersal (Graystock et al., 2015), mass‐flowering crops could facilitate pollinator parasites dispersal. Therefore, understanding the impact of pollinator parasites on plant–pollinator interactions could lead to the development of useful mitigation strategies in agriculture.

## 7 CONCLUSION

The significance of pollinator‐mediated selection makes it a major driving force of the evolution and diversification of flowering plants (Fenster et al., 2004; Sapir & Armbruster, 2010). Therefore, variations in pollinator foraging behaviors are essential to understanding the evolutionary mechanisms at play in plant–pollinator interactions, especially in case of pollen limitation (Ashman et al., 2004; Knight et al., 2005). Pollinator parasites are readily transmitted among pollinators through shared‐use of flowers (Graystock et al., 2015). In response to parasites, pollinators have adapted altered foraging behaviors (Fouks & Lattorff, 2011; Richardson et al., 2016), which can affect flower choice and may influence pollen dispersal. Therefore, pollinator parasites may indirectly affect plant fitness, especially if parasites trigger immune behaviors in most or all pollinators species that efficiently pollinate that plant. Floral traits could be selected to minimize the impact of pollinator parasites on plant fitness, with strong selection on floral traits acting against several pollinator parasites and upon several immune behaviors. For example, a bacteria infecting several pollinators, such as the insect parasite *Paenibacillus alvei* (Sadd & Schmid‐Hempel, 2006; Grady et al., 2016), could exert strong selection on plants relying on insect pollinators for their reproduction. Such bacteria could be deposited on flowers by inefficient pollinators, limiting the probability of successful pollination. Once deposited, these bacteria could repel pollinators and thus reduce plant fitness. In such case, the increased secretion of certain compounds in nectar, such as antimicrobial compounds present in scented nectar (Raguso, 2004), capable of diminishing bacterial load and masking bacterial presence, could limit the efficiency with which pollinators exhibit disease avoidance. Moreover, antimicrobial compounds in nectar may increase visitation by self‐medicating pollinators, and could thus lead to increased plant fitness.

Plants evolve through the action of several selective forces: abiotic factors (e.g., drought, wind) and biotics factors (e.g., herbivores, pollinators, robbers, competition). Those selective forces can act in synergy or antagonistically, depending on floral traits, leading either to strong selection or balancing selection. While pollinator parasites vary in prevalence through space and time, and therefore may not represent a major force of selection in flowering plants on their own, they can act in synergy or antagonistically with other selective forces in significant ways. In this context, pollinator parasites may be as important as to other selective forces, strengthening or weakening the selection direction of certain floral traits. In order to better understand the evolution of flowers and floral diversity, the selective forces acting on the plants, as well as their interactions, need to be more comprehensively identified and described. By influencing pollinator foraging behaviors, pollinator parasites may serve as an important, and previously underestimated selective force in the evolution of plant–pollinator interactions and floral diversity.

## CONFLICT OF INTEREST

None declared.

## AUTHOR CONTRIBUTION

BF performed all analyses and wrote the first draft of the manuscript, BF & KMW contributed substantially to revisions; viewed and commented on the last draft.

## GLOSSARY

**Table:** 

Parasites	All organisms that, either during some or all stages of their life‐cycle, feed on another organism without resulting in the immediate death of their host. This definition includes viruses, bacteria, fungi, protozoa and arthropods, as well as endo‐ and ecto‐parasites and parasitoids.
Behavioral immunity/Behavioral Immune System	All modified animal behaviors adapted to enhance resistance and/or tolerance against parasites, including behaviors adapted to limit parasite uptake, intake, establishment, spread, transmission and impact on host fitness.
Self‐medication	Any therapeutic and prophylactic behavior that is related to contact with or consumption of biologically active chemicals, and which results in the reduction or elimination of parasitic infection or related symptoms (Abbott, 2014).
Disease Avoidance/Behavioral Avoidance	A spatial, temporal, or trophic modulation of behavior preventing infection or reducing infective doses by limiting contact and uptake of parasites. In other words, any behaviors resulting in physical avoidance of parasites before contact is made (thus excluding grooming behavior).
Immune grooming	Grooming is defined as any act related to the maintenance of one's own body surface or the body surface of a conspecific. Here, immune grooming refers to grooming behaviors triggered by parasites, which increase the likelihood of the removal of parasites from one‧s own body surface or the body surface of a conspecific.
Trait‐mediated indirect interactions	The modifications of interactions between two species due to a change in behavior, physiology, or morphology of a third species.
Density‐mediated indirect interactions	The modifications of interactions between two species due to a change in population density of one species through the impact of a third species.
Foraging	All behaviors associated with the search for and collection of food. (Note that in Figure 1, we disentangle the search and collection of food, where “foraging” refers to all foraging behaviors except feeding. We made this distinction since flowering plants use two types of traits to attract pollinators: rewards (or “primary attractants”) and advertisements (“secondary attractants”) (Fenster et al., 2004). Rewards constitute the primary or economic motivation for animals to visit flowers, while advertisements attract the attention of pollinators and promote associative learning).

## Data Availability

No data.
