# Supplementary figures and images for: Pollinator parasites and the evolution of floral traits
Source: Ecol Evol. 2019 May 15;9(11):6722–37. doi: 10.1002/ece3.4989 (PMC6580263; doi:10.1002/ece3.4989)

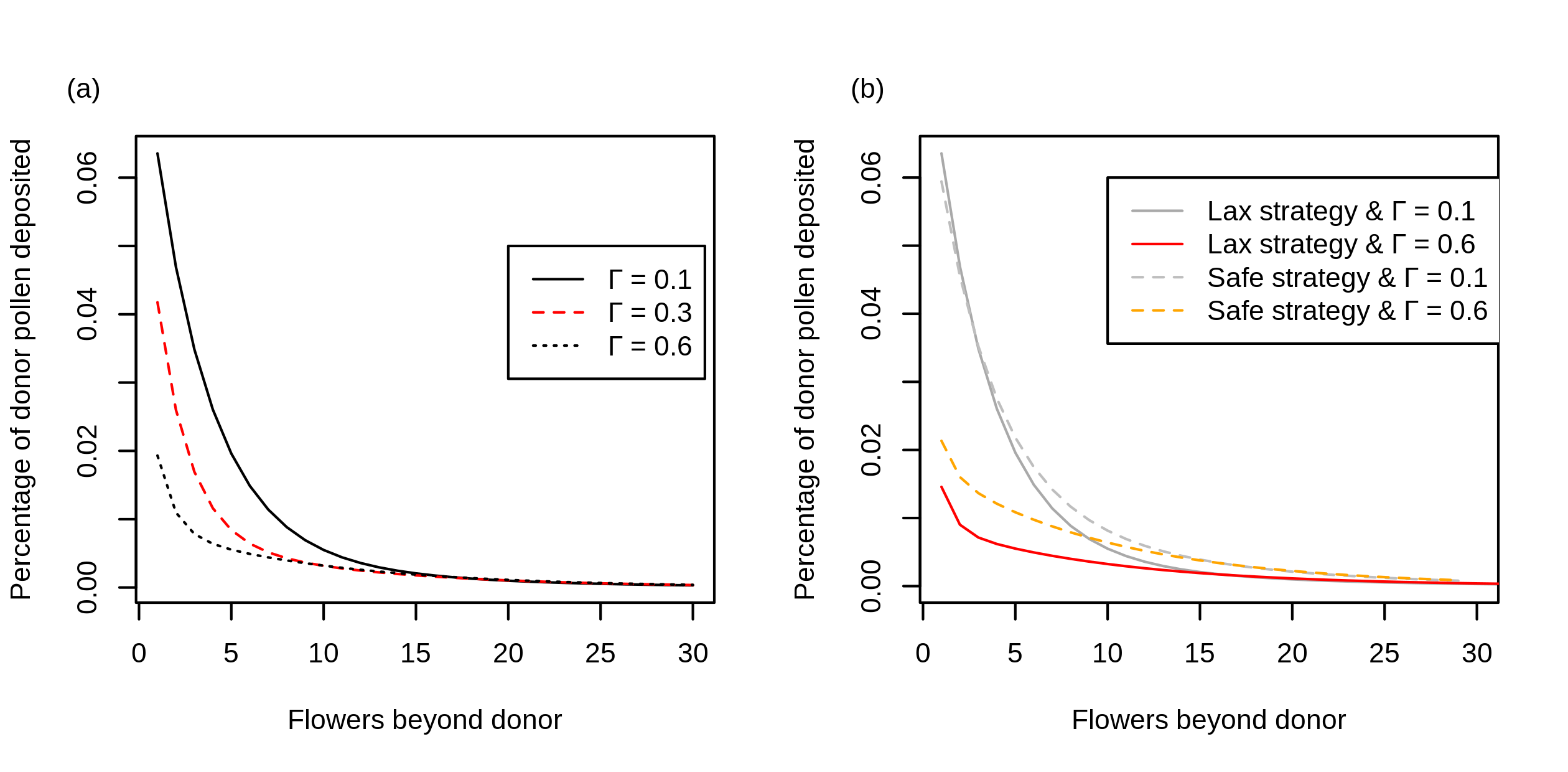

Supplement: Supplementary file 1 [file ECE3-9-6722-s001.tif]
