# Supplementary material for: Pollinator parasites and the evolution of floral traits
Source: Ecol Evol. 2019 May 15;9(11):6722–37. doi: 10.1002/ece3.4989 (PMC6580263; doi:10.1002/ece3.4989)
Supplement: Supplementary file 2 [file ECE3-9-6722-s002.docx]

**Figure S1**: Pollen dispersal models with varying grooming intensities. (a) Model of pollen dispersal in relation to grooming intensity. (b) Model of pollen dispersal in relation to grooming intensities and flower pollen dispersal strategies. R = 100, π_s_ = 0.05, π_e_ = 0.45, π = 0.5, *у_s_* = 0.1, ρ_e_ = 0.2, ρ_s_ = 0.1, grooming intensity: Г, safe strategy: π_e_ = 0.35, π_s_ = 0.15; 70/30, and lax strategy: π_e_ = 0.45 and π_s_ = 0.05; 90/10.
